# Nuclear Mechanisms Involved in Endocrine Resistance

**DOI:** 10.3389/fonc.2021.736597

**Published:** 2021-09-15

**Authors:** Jürgen Dittmer

**Affiliations:** Clinic for Gynecology, Martin Luther University Halle-Wittenberg, Halle, Germany

**Keywords:** fulvestrant, tamoxifen, estrogen receptor, transcription factors, chromatin accessibility, transcriptional reprogramming, cancer stem cells

## Abstract

Endocrine therapy is a standard treatment offered to patients with ERα (estrogen receptor α)-positive breast cancer. In endocrine therapy, ERα is either directly targeted by anti-estrogens or indirectly by aromatase inhibitors which cause estrogen deficiency. Resistance to these drugs (endocrine resistance) compromises the efficiency of this treatment and requires additional measures. Endocrine resistance is often caused by deregulation of the PI3K/AKT/mTOR pathway and/or cyclin-dependent kinase 4 and 6 activities allowing inhibitors of these factors to be used clinically to counteract endocrine resistance. The nuclear mechanisms involved in endocrine resistance are beginning to emerge. Exploring these mechanisms may reveal additional druggable targets, which could help to further improve patients’ outcome in an endocrine resistance setting. This review intends to summarize our current knowledge on the nuclear mechanisms linked to endocrine resistance.

## Introduction

Breast cancer (BC), a systemic disease characterized by early tumor cell dissemination (1), is the most frequent cancer among women and leading cause of cancer-related death in women worldwide (2). Disseminated BC cells often enter dormancy and may later grow out to a metastatic lesion (3, 4). In a metastasis-free state, there are good therapy options to substantially prolong survival of BC patients. BC is a heterogenous disease, requiring subtyping, classically based on immunohistochemistry (IHC), to offer the patient the best possible treatment. The statuses of estrogen receptor α (ERα), progesterone receptor (PR) and human epidermal growth factor receptor 2 (Her2) are routinely examined. The majority of BCs are ERα/PR-positive. Additionally, Her2-positive BCs and triple-negative (ERα-, PR- and Her2-negative) BCs (TNBCs) are found. Subtyping by mRNA expression profiling revealed four major BC subtypes (luminal A, luminal B, Her2-enriched and basal-like) (5), which overlap with the IHC-subtypes. Luminal A and B tumors are mostly ERα-positive BCs, whereby luminal B tumors are more aggressive. Basal-like BCs show commonly features of TNBCs.

Routine treatment options for BC patients include ERα- and Her2-targeting therapies, chemotherapy, surgery and radiation. Besides ERα and Her2 expression, the luminal subtype, tumor grading and lymph node involvement play a role in therapy decision (6). Endocrine therapy is a standard treatment for patients with ERα-positive BCs. Two principal strategies are used in endocrine therapy to block estrogen-dependent ERα activity. One strategy utilizes anti-estrogens to compete with estrogen for binding to the ERα protein. Anti-estrogens are roughly divided in selective ERα modulators (SERMs), such as tamoxifen (TAM) (7, 8), and selective ERα downregulator (SERDs), such as fulvestrant (FULV) (9, 10). In contrast to SERMs, SERDs are pure ERα inhibitors, induce ERα degradation and prevent ERα from becoming transcriptionally active (9–11). In the other strategy, estrogen synthesis is blocked by an aromatase inhibitor (AI), such as exemestane, resulting in estrogen deficiency (12). Both strategies are effective for treating ERα-positive BCs.

Endocrine resistance (ENDO-R), the resistance to ERα-targeting therapy, is a major obstacle in treatment of ERα-positive BCs. In first-line treatment, ENDO-R is observed in approximately half of all ERα-positive BCs (13). Many factors contributing to ENDO-R have been identified. While there are excellent reviews on the mechanisms of endocrine resistance, which primarily focus on signaling pathways, cell cycle regulators, microRNAs and/or mutation in the ERα-coding gene *esr1* (14–19), this review preferentially aims to summarize the currently known nuclear mechanisms that contribute to ENDO-R. Where necessary, event(s) in other cellular compartments that are crucially linked to the nuclear mechanism discussed, are also described.

## ERα, the Target of Endocrine Treatment

### The ERα Protein

There are two estrogen receptors, ERα and ERβ (20). While ERβ is generally considered to act anti-proliferative, ERα promotes proliferation. Estrogen-activated ERα is a potent stimulator of cyclin D1 expression (21, 22), leading to activation of cyclin-dependent kinases (CDKs) 4 and 6, which in turn phosphorylate retinoblastoma protein to initiate cell cycle entry (23).

Expression of ERα is regulated by transcription of its gene estrogen receptor 1 (*esr1*) and by proteasome-dependent degradation of the ERα protein (24). Primarily, ERα acts as a transcription factor on estrogen-responsive element (ERE)-containing genes by directly binding to its recognition sequence. It is also possible that ERα binds indirectly to DNA by tethering to other transcription factors, such as activating protein-1 (AP-1) (25, 26). Two trans-activation domains, transactivation function (AF)-1 and AF-2, allow ERα to interact with the transcriptional machinery, whereby AF-2 mediates estrogen-dependent ERα transcriptional activity (27). Two splice variants of ERα, ERα46 and ERα36, exist, whereby ERα46 does not contain the AF-1 domain and ERα36 lacks both transactivation domains.

The ERα protein can be phosphorylated at many sites, which has an impact on its activity (27). Particularly important are phosphorylations at Ser-118 and Ser-167 in the AF-1 domain. These modifications, which promote ligand-dependent as well as ligand-independent transcriptional activities of ERα, affect the interaction of ERα with transcriptional co-factors, such as CREB (cAMP regulatory element binding protein)-binding protein (CBP) or steroid receptor co-activator (SRC). Phosphorylation at these sites can be triggered by receptor tyrosine kinases (RTKs) through the PI3K/AKT/mTOR/p70S6K and the Ras/Raf/MEK1/ERK1/2 pathways.

Besides genomic activities, non-genomic activities of ERα have been documented, which leads to the activation of the PI3K/AKT/mTOR/p70S6K and the Ras/Raf/MEK1/ERK1/2 pathways (27). These activities may involve interactions of ERα with PI3K and the non-receptor tyrosine kinase c-Src.

### Role of ERα in Endocrine Resistance

Given that ERα is the key transcriptional driver in ERα-positive BC cells, it is not surprising that ERα inhibitors have a tremendous effect on transcription. Exposure of MCF-7 cells to anti-estrogens leads to altered expression of approximately two-thirds of 1.8 x 10^4^ studied genes (28). Though TAM or FULV induce similar changes in gene expression, it takes different strategies to overcome the inhibitory actions of the two anti-estrogens. In the presence of TAM, ERα can still be active in an estrogen-independent manner allowing ERα-based escape mechanisms. Indeed, one study showed that ERα was transcriptionally active in approximately three quarters of BC specimens from patients who relapsed on TAM (29). ERα-based escape mechanisms in TAM resistance include phosphorylation of the ERα protein, overexpression of ERα co-activators, such as SRC-1, a switch to AP-1-responsive gene activation and a shift from genomic to non-genomic ERα activities (25, 30). Nevertheless, as shown with MCF-7 cells, TAM resistance coincides with an altered chromatin organization (31). In addition, hundreds of genes are differently expressed in tamoxifen resistant (TAM-R) cells as compared to estrogen-treated parental cells.

Like TAM resistance, resistance to AI often occurs with the ERα protein remaining active. A frequent escape mechanism involves a mutation in the AF-2 domain allowing constitutive ERα activation in the absence of estrogen (16, 18).

As pure ERα antagonists, SERDs block ERα activity completely (10), requiring cells to find escape routes independent of ERα usage. In fact, in clinical samples, FULV resistance is associated with decreased ERα pathway activity (32). Also, in contrast to TAM-R MCF-7 cells, fulvestrant resistant (FULV-R) MCF-7 cells show almost no response of ERα-regulated genes to estrogen (33). Furthermore, TAM-R sublines are usually sensitive to FULV (34). Nevertheless, in some cases, TAM resistance may be accompanied by FULV resistance (34) suggesting that TAM resistance can also be achieved by ERα-independent mechanisms.

### Pre-existing *vs.* Acquired Endocrine Resistance in Established BC Cell Lines

Drug resistance can either happen when cells in the tumor pre-exist that are intrinsically insensitive to the drug or when cells acquire resistance during treatment. Given the heterogenous nature of tumors (35, 36), it is not unlikely that drug-resistant cell clones have spontaneously developed during clonal evolution (37) without having been challenged by a particular drug. Such pre-existing drug-resistant clones would be expected to allow the cancer to rapidly progress under treatment pressure. In fact, a study on patients with ERα-positive advanced BC treated with FULV and the CDK4/6 inhibitor palbociclib revealed that cancers with pre-existing escape mutations reduced progression free-survival significantly and showed “no need” to develop additional escape mutations (38).

Most of our knowledge on mechanisms underlying anti-estrogen resistance has come from studies with established BC lines, predominantly MCF-7, T47D and ZR75-1. These cell lines have been established from pleural effusions of metastatic BC patients (39). Their ERα chromatin binding profiles overlap with those of primary BCs with poor outcome confirming the aggressive nature of these cell lines (40). Numerous studies demonstrated that the MCF-7 cell line is a heterogenous population (41–45), which, when challenged by anti-estrogens, form multiple FULV-R and TAM-R clones, all containing the same DNA aberrations (46). This suggests that the resistant clones all derived from one subpopulation of cells that pre-existed in the MCF-7 cell line (46). It would explain why FULV-R clones appear rapidly (within a couple of weeks) when MCF-7 cells are exposed to FULV (45). Importantly, at the time when MCF-7 or other commonly used BC lines were established, endocrine therapy was not available (40). Hence, it is likely that established BC cell lines contain cells that spontaneously became endocrine resistant in the absence of endocrine treatment before the tumor cells have been collected from the patient decades ago. This should be taken into consideration when interpreting the results obtained in resistance studies with established BC cell lines.

### Current Targets for Therapy in Endocrine Resistance

The PI3K/AKT/mTOR pathway has become a major focus in ENDO-R research and has stimulated the development of drugs that target this pathway (47, 48). PI3K and mTOR inhibitors have been found to be effective drugs to treat patients with an endocrine resistant BC (49, 50). More recently, CDK4 and CDK6 have been shown to be appropriate druggable targets in ENDO-R (17, 51). Combinatorial treatments with drugs directed to the PI3K/AKT/mTOR pathway and to CDK4/6 are discussed to further improve treatment efficacy (52).

**Figure 1:**
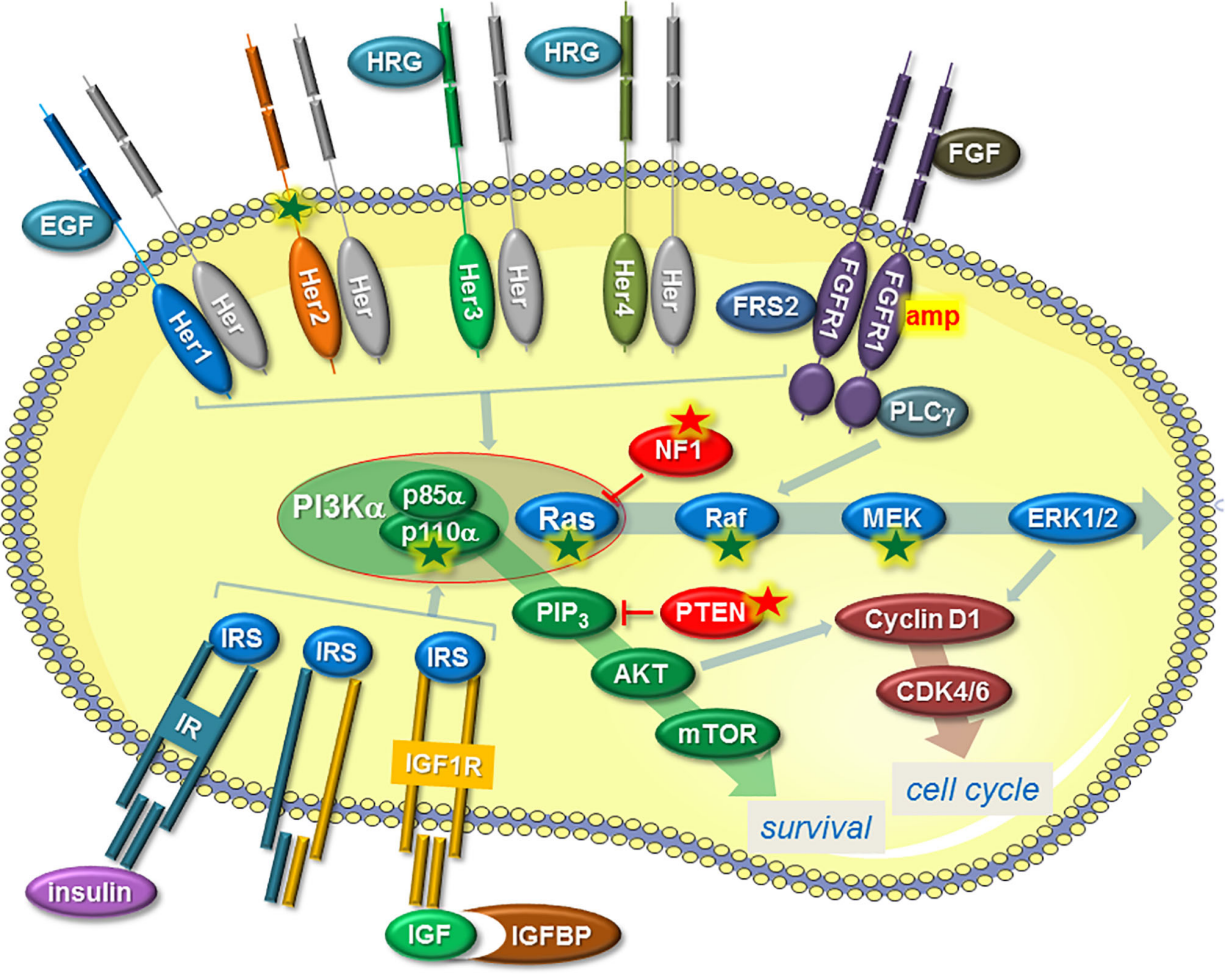
Mechanisms that induce ENDO-R by deregulation of the PI3K/AKT/mTOR and/or Ras/Raf/MEK/ERK1/2 pathways, two pathways that not only stimulate proliferation by raising cyclin D expression and thereby activating CDK4/6 but also promote survival. A common mechanism involves a higher activity of certain RTKs. This includes higher activities of Her proteins, induced by higher availability of ligands, such as EGF or HRG, or by gain-of-function mutation (Her2). FGFR1 is often amplified (amp) in ENDO-R and requires co-factors FGFR substrate 2 (FRS2) and phospholipase C-γ; (PLC-γ) to activate the two pathways. IR or IGF1R may contribute to ENDO-R if activated by insulin or IGFs. Higher IGF availability can be achieved by reduced expression of IGF binding proteins (IGFBPs). The expression of the IR/IGF1R co-factor insulin receptor substrate (IRS) may also play a role in ENDO-R. RTK-independent activation of PI3K/AKT/mTOR pathway is commonly caused by a gain-of-function mutation of the gene *pik3ca* coding for p110α, which together with p85α forms the PI3Kα complex. Dysfunction of PTEN, which prevents AKT activation by blocking the formation of phosphatidylinositol-3,4,5-trisphosphate (PIP_3_) is another way by which this pathway can be upregulated. RTK-independent activation of the Ras/Raf/MEK/ERK1/2 pathway in ENDO-R include gain-of-function mutations in *ras*, *raf* or *mek*-encoding genes as well as dysfunction of NF1, an inhibitor of Ras. Arrows indicate positive, T-shaped symbols negative effects. A green or red star denotes a gain-of function or a loss-of-function mutation/deletion, respectively.

Activation of the PI3K/AKT/mTOR pathway in ENDO-R can occur in different ways and most often involves RTKs, including epidermal growth factor receptor (EGFR), Her2, Her3, Her4, fibroblast growth factor receptor (FGFR), insulin-like growth factor receptor (IGF1R) and insulin receptor (IR) (**Figure 1**). Other ways are a gain-of-function mutation in the phosphatidylinositol-4,5-bisphosphate 3-kinase catalytic subunit alpha *(pik3ca*) gene coding for the PI3K catalytic component p110α (53, 54) or a loss of phosphatase and tensin homolog (PTEN) (55). RTKs are often deregulated in ENDO-R by overexpression (EGFR, Her2, Her3, Her4) (34, 56–58), partly as a result of gene amplifications (FGFR1) (59, 60), by mutations (Her2) (61) or by higher availability of RTK ligands, such as heregulin, IGF1 or insulin (Her3, Her4, IGF1R, IR) (62–64).

As the second major pathway that is activated by RTKs, the Ras/Raf/MEK/ERK1/2 pathway also contributes to ENDO-R (**Figure 1**). Independent of RTKs, this pathway can also be activated by mutations in Ras, Raf or MEK or by downregulation of the Ras inhibitor neurofibromatosis type 1 (NF1) (61).

Dual CDK4/6 inhibitors, such as palbociclib (PD-0332991), in combination with endocrine therapy are currently standard of care for advanced ERα-positive breast cancer (51, 65–68). Activation of CDK4/6 requires physical interaction with their co-factor cyclin D1 (69), whose level raises upon activation of certain proteins, such as RTKs or ERα (70). High expression of cyclin D1 is associated with poor prognosis in ERα-positive breast cancer (71) and linked to an increased risk of relapse on TAM (72). In FULV resistance, of the two CDKs particularly CDK6 may play a role. Increased expression of CDK6 was reported in FULV-treated MCF-7 cells (73, 74). Inhibition of CDK6 suppressed growth of FULV-resistant MCF-7 cells. A high CDK6 level in breast cancer of FULV-treated metastatic patients was found to predict a worse outcome (74).

Deciphering the changes happening in the nucleus upon acquisition of ENDO-R may result in the identification of additional druggable factors in ENDO-R.

## Transcription Factors

**Figure 2:**
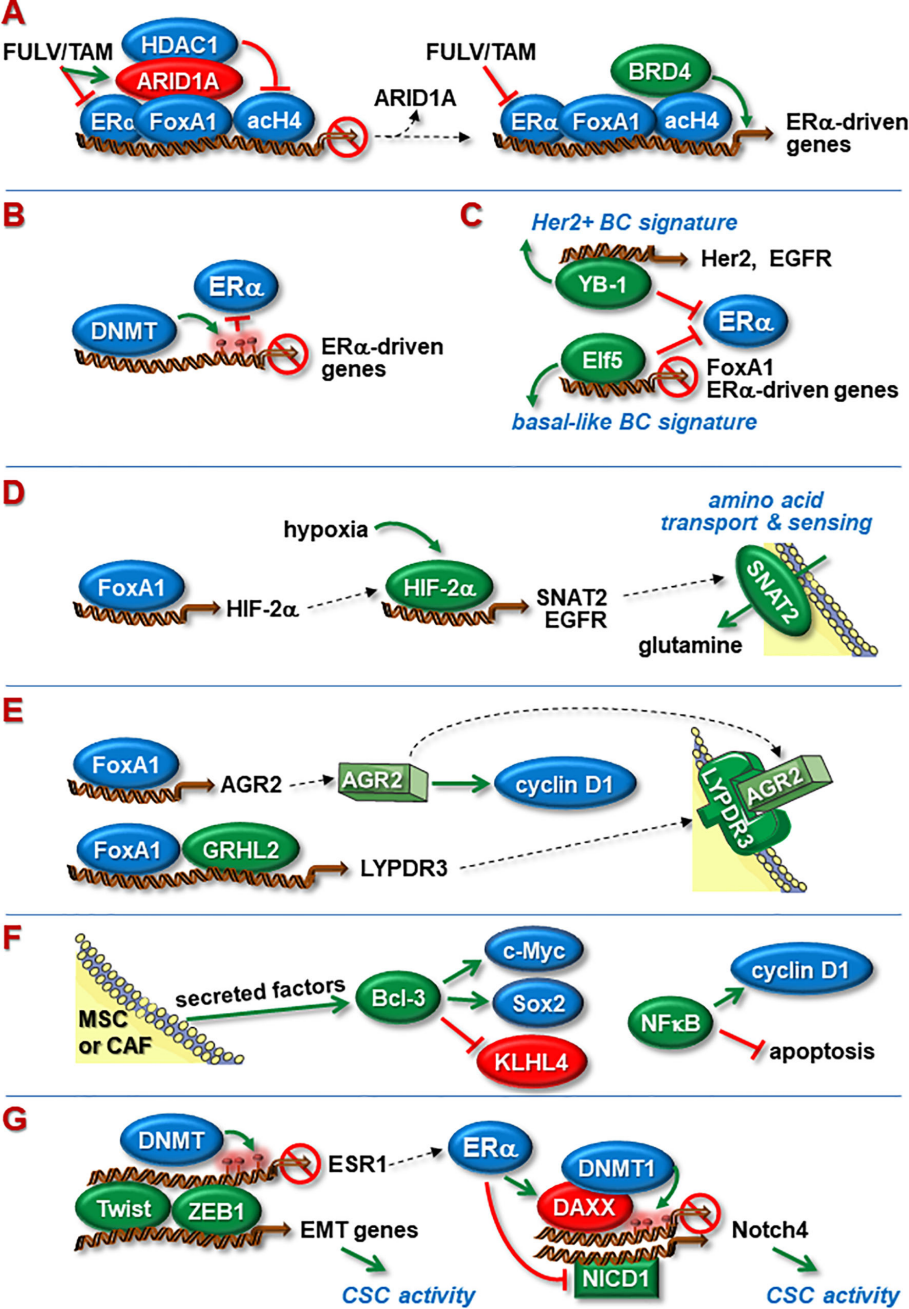
Nuclear proteins involved in ENDO-R. **(A)** Blockage of ERα function by FULV or TAM causes ARID1A to bind to FoxA1 leading to transcriptional inhibition of ERα-driven genes by recruitment of HDAC1. Dysfunctional ARID1A leads to higher abundance of acetylated histone 4 (acH4) and recruitment of BRD4, able to active transcription despite the presence of anti-estrogens. **(B)** ENDO-R often coincides with DNMT-mediated DNA methylation of ERα-driven genes at promoters and/or enhancers, resulting in blockage of ERα binding to these sites. **(C)** Acquisition of ENDO-R by transcriptionally re-programming cells. YB-1 suppresses ERα activity and upregulates the expression of Her2 and EGFR leading to a Her2-driven transcriptional pattern. Elf-5 inhibits the expression of ERα and FoxA1 and fosters a transcriptional pattern typically seen in basal-like breast cancer. **(D)** Hypoxia promotes ENDO-R by activating HIFs. FoxA1-regulated HIF-2α stimulates the transcription of EGFR and SNAT2, the latter being a transmembrane transporter and sensor of amino acids. Anti-estrogen resistant cells may use SNAT2-imported glutamine as a major carbohydrate source to maintain metabolism. **(E)** Independent of ERα, FoxA1 can stimulate the transcription of AGR2 and, in cooperation with GRHL2, the transcription of LYPDR3. AGR2 can cause the cyclin D1 synthesis to rise. FoxA1, GRHL2, LYPDR3 and AGR2 may act in concert to induce ENDO-R. **(F)** Members of the NFкB/Iк;B family may be involved in ENDO-R. NFкB supports ENDO-R by stimulating cyclin D1 expression and by inhibiting apoptosis. Bcl-3, whose expression in BCs is induced by MSC- and CAF-secreted factors, causes higher expression of proliferation-stimulatory c-Myc and anti-apoptotic stem cell factor Sox2 and blocks proliferation-inhibitory KLHL4. **(G)** Twist and ZEB1 can enhance CSC activity by inducing EMT. Additionally, Twist and ZEB1 can suppress ERα expression by recruiting DNMT to the *esr1* promoter. ERα may limit CSC activity by suppressing the transcription of Notch4. One way involves induced expression of the transcriptional repressor DAXX followed by DNMT1-dependent methylation, another down-regulated abundance of Notch1-derived NICD1, a positive regulator of Notch4 transcription. Green and red ovals indicate proteins that promote or inhibit anti-estrogen resistance, respectively. Green arrows indicate a positive, red T-shaped symbols a blocking effect. Red circles denote CpG methylations.

Sequence-specific transcription factors (TFs) are key drivers of gene expression and can have activating or repressive functions. Activating TFs induce gene transcription by binding to promoters and/or enhancers followed by recruitments of co-activators and RNA polymerase (75). Two major types of activating TFs are distinguished: pioneer and settler TFs (76). Pioneer TFs assist loading of settler TFs by initiating chromatin accessibility (*Chromatin Accessibility*). Both types of TFs are involved in ENDO-R (**Figure 2**). They may act as effectors of signaling pathways involved in ENDO-R and/or may reprogram cells from ERα-dependent to ER-independent gene expression.

### AP-1

ATF2 and c-Jun are members of the AP-1 family of transcription factors and often form heterodimers (77, 78). Impairment of ERα activity can lead to a shift from ERE-dependent to AP-1-dependent ERα-induced transcription, involving c-Jun (79). Interestingly, c-Jun activity can be regulated by RTKs, partly through the Ras/Raf/MEK/ERK1/2 and the PI3K/AKT signaling pathways (80) linking c-Jun to RTK-induced ENDO-R. Another study showed that silencing of ATF2 in FULV-R and TAM-R MCF-7 sublines strongly decreased ERα-independent cellular growth and concomitantly increased the level of ERα and the expression of ERα-responsive genes (81). On the other hand, ATF-2 and c-Jun can have opposite effects on genes. For instance, while c-Jun represses, ATF-2 activates PTEN transcription in BC cells, leading to opposing effects of the two AP-1 members on AKT activity (82).

A screen in a chemical library resulted in the discovery of two ATF-2 inhibitors, celastrol (CSL) and acetyl isogambogic acid (AIGA), which both proved to be potent inhibitors of melanoma growth (83). CSL, known as an anti-inflammatory drug, could also be shown to counteract cis-platin resistance of non-small cell lung cancer by inhibiting ATF-2 (84).

### E74-Like Factor 5

Elf5, also known as epithelium-specific Ets transcription factor 2 (ESE2), a member of the E26-transformation-specific/E-twenty-six-specific sequence (ETS) domain family of transcription factors (85), plays a role in BC progression (86). Elf5 is highly expressed in basal-like BCs, while its expression in luminal BCs is lower than in normal breast tissue (87). However, resistance of MCF-7C cells to FULV and TAM coincides with an increase in Elf5 expression. Ectopic expression of Elf5 in MCF-7 and T47D cells was found to down-regulate ERα and FoxA1 levels and to suppress the expression of ERα-driven genes. Additionally, it induces a gene signature resembling that of basal-like BC cells. It is thought that the Elf5-induced switch from a luminal to a basal-like subtype may be one route for ERα-positive BC cells to escape the growth-suppressing effects of anti-estrogens.

### Estrogen-Related Receptor-α

ERRα is an orphan nuclear receptor that shows a high homology to ERα in the DNA binding domain, potentially allowing ERRα to activate ERα-target genes in the absence of estrogen (88). Higher levels of ERRα and lower levels of ERα were found in BC specimens from patients who relapsed on TAM compared to BC specimens from untreated patients (29). Furthermore, in TAM-R and FULV-R MCF-7 cells, ERRα expression is increased, while silencing of ERRα has a stronger inhibitory effect on growth of resistant sublines than it does on the growth of the parental cell line. Moreover, high ERRα expression predicts poor prognosis for TAM-treated patients (29) (**Table 1**).

**Table 1:** Nuclear proteins linked to endocrine resistance and their impacts on clinical outcome in breast cancer.

Protein	Cohort	N (patients)	Molecule analyzed	Detection method(s)	Prognosis	Independent marker in multivariate analysis?	Reference
***ARID1A***	pat. w/primary BC	476	protein	IHC	high ARID1A ⇨ higher DFS and OS (all BCs, lum A)	Yes, indicative for good outcome	(89)
***ARID1A***	pat. w/BC	1824	DNA	mutational status	mutant ARID1A ⇨ lower OS	n.a.	(28)
***Bcl-3***	pat. treated w/TAM only	229	mRNA	KM-P (*in silico*)	higher Bcl-3 ⇨ lower RFS	n.a.	(90)
***DAXX***	pat. treated w/ET only or received NST	742 (ET) 503 (NST)	mRNA	KM-P (*in silico*)	high DAXX ⇨ higher RFS (ET), high/low DAXX⇨ same RFS (NST)	n.a.	(91)
***ERRα***	pat. treated w/TAM	239 dataset GSE9893	protein mRNA	IHC MA (*in silico*)	high ERRα ⇨ lower OS	Yes (mRNA and protein), indicative for poor outcome of TAM-treated pat.	(29)
***FoxA1***	pat. treated w/TAM only or w/o ET	615 (TAM)	mRNA	KM-P (*in silico*)	high FoxA1 ⇨ lower RFS (TAM) high/low FoxA1 ⇨ same RFS (no ET)	n.a.	(92)
		500 (no ET)					
***FoxA1***	pat. treated w/ET only or received NST	997 (TAM)	protein	IHC	high FoxA1 ⇨ high RFS (TAM and NST)	Yes, indicative for good survival of pat. w/ERα-pos. BC	(93)
***FoxM1***	pat. w/BC	965 (lum A)	mRNA	KM-P (*in silico*)	high FoxM1 ⇨ lower DMFS (lum A, B) lower RFS (TAM)	n.a.	(94)
		430 (lum B)					
		809 (TAM only)					
***H2A.Z***	pat. w/BC	517	protein	IHC	high H2A.Z ⇨ lower OS	Yes, indicative for poor outcome	(95)
***HDAC***	pat. who relapsed on ET (HDACi + exe *vs.* placebo + exe)	365	none	none	HDACi ⇨ higher PFS	n.a.	(96)
***Notch***	pat. w/ERα-pos. BC	1862	mRNA	KM-P (*in silico*)	high Notch activity ⇨ lower RFS and DMFS	n.a.	(97)
***Notch***	pat. treated w/TAM or received NST	669 (TAM)	mRNA	MA data sets (*in silico*)	high Notch activity ⇨ lower DMFS (TAM), lower OS (NST)	n.a.	(98)
		343 (NST)					
***Snail Slug Twist***	pat. w/non-metastatic BC	289	protein	IHC	high Snail, Slug or Twist ⇨ lower RFS	Yes (Snail and Twist combined), indicative for poor survival of pat. w/ERα-pos. BC	(99)
***XBP1***	pat. w/ERα-pos. BC	97	mRNA	Q-RT-PCR	high XBP1(U) ⇨ higher RFS high XB1(S/U) ratio ⇨ lower RFS	Yes, XB1(S/U) ratio indicates poor survival of pat. w/ERα-pos. BC	(100)
***YB-1***	pat. w/newly diagnosed invasive BC	4049	protein	IHC	high YB-1 ⇨ lower BCSS (all BCs, TAM treatment)	Yes, indicative for poor outcome	(101)

BC, breast cancer; BCSS, breast cancer-specific survival; DFS, disease-free survival; DMFS, distant metastasis-free survival; ET, endocrine treatment; HDACi, HDAC inhibitor; IHC, immunohistochemistry; KM-P, Kaplan-Meier plotter (http://kmplot.com/analysis); MA, cDNA microarray; n.a., not analyzed; OS, overall survival; pat., patients; PFS, progression-free survival; Q-RT-PCR, quantitative reverse transcription polymerase chain reaction; RFS, relapse-free survival; TAM, tamoxifen; NST, no systemic treatment.

The potential role of ERRα in diabetes has encouraged the development of ERRα inhibitors (102). Specific ERRα-targeted drugs have been generated by preventing the recruitment of the co-activator SRC to the ERRα protein. These drugs were proven to have little effects on ERRα relatives ERRβ and ERRγ, did not influence ERα activity and were well tolerated when administered to rats. Such inhibitors were also shown to act anti-proliferatively on breast cancer cells *in vitro* and *in vivo* (103). They may be potentially useful for treating ERRα-induced ENDO-R.

### Forkhead Box Protein A1

Expression of FoxA1 correlates with ERα expression in primary ERα-positive BCs (104). As a pioneer TF, FoxA1 facilitates ERα binding to promoters and enhancers and cooperates with ERα to drive ERα-dependent transcription (105). Most of the ERα binding takes place outside of proximal promoters (106), coinciding with enhanced gene looping allowing recruitment of distal regulatory transcriptional machinery (31). Silencing of FoxA1 results in failure of estrogen to stimulate growth of MCF-7 or ZR75-1 cells, confirming the essential role of FoxA1 in ERα function (105).

Overexpression of FoxA1 leads to transcriptional reprogramming mainly based on higher FoxA1 occupation of so-called super enhancers (107). Super enhancers are clusters of enhancers densely occupied with transcription factors and located in the vicinity of genes important for cell identity (108). Ectopic expression of FoxA1 desensitizes MCF-7 cells to FULV and TAM (92). Furthermore, FoxA1 was found to be overexpressed in TAM-R sublines derived from MCF-7 and BT474 (92), though TAM resistance of MCF-7 cells may also coincide with a lower FoxA1 level (109). FoxA1 is frequently overexpressed in primary BC, which happens more often in luminal B than luminal A tumors (92).

There are contradicting results in terms of the predictive value of FoxA1 overexpression for TAM-treated patients. While higher FoxA1 mRNA levels correlated with poor survival (92), FoxA1 protein overexpression was associated with favorable outcome (93). In 3.7% of primary BCs and even in 7% of lobular BC the FoxA1 gene is mutated (110). These mutations were found to be associated with higher FoxA1 expression and activity.

Among the genes targeted by FoxA1 is anterior gradient 2 (AGR2), the human homologue of XAG-2, a *Xenopus laevis* protein playing a potential role in neural development (111). AGR2 is a protein disulfide isomerase and involved in protein maturation control in the endoplasmic reticulum (112). In murine mammapoiesis, AGR2 regulates epithelial proliferation and lobuloalveolar development (113). AGR2 is able to upregulate the EGFR ligand amphiregulin (114) and the expression of cyclin D1 (115), being consistent with the finding that, in primary BC, the level of cyclin D1 correlates with that of AGR2 (116).

As shown with MCF-7 cells, AGR2 is important for ERα-driven proliferation (112, 116–120). AGR2-overexpressing MCF-7 cells show a delay in FULV-induced ERα degradation, likely caused by physical interaction of the AGR2 with the ERα protein (121). Silencing of AGR2 increased the sensitivity of ZR75-1 and T47D cells to FULV and TAM, reduced c-Src kinase activity and decreased the level of the anti-apoptotic protein survivin (115). In TAM-R MCF-7 cells, AGR2 is highly expressed while being mainly regulated by FoxA1 independently of ERα (117). If secreted, AGR2 can bind to the membrane receptor LY6/PLAUR domain containing 3 (LYPD3), whose expression is regulated by FoxA1 in cooperation with the transcription factor grainyhead like transcription factor 2 (GRHL2) (122). There is evidence that AGR2, LYPD3, GRHL2 and FoxA1 act together to foster ENDO-R.

Higher AGR2 expression is associated with unfavorable prognosis in BC (117). This holds true also for ERα-positive BC (116), where AGR2 is more abundant (112, 123). Furthermore, higher AGR2 expression predicts a weaker response to TAM in primary BC (116, 122).

Antibodies against AGR2 and LYPD3 have been found to be effective to suppress growth of TAM-R breast cancer cells in mice (122). Additionally, humanized anti-AGR2 and anti-LYPD3 antibodies are in development. In a pre-clinical trial, an anti-LYPD3 antibody-auristatin conjugate (BAY 1129980) is tested for treatment of LYPD3-expressing non–small cell lung cancer (124).

### FoxM1

The FoxM1 gene is transcriptionally regulated by ERα and is important for ERα-driven cellular growth (125). Accordingly, FULV and TAM reduce FoxM1 expression. However, long-term treatment with TAM increases FoxM1 expression in MCF-7 cells (126), while FoxM1 depletion sensitizes TAM-R MCF-7 cells to TAM (125). Among the genes upregulated by FoxM1 are cyclin D1 and ATP-binding cassette super-family G member 2 (ABCG2) (125, 126). ABCG2, a transporter protein that pumps drugs out of the cell (127), was found to contribute to anti-estrogen resistance (126). Many genes, including ABCG2, require active ERK2 for FoxM1-dependent transcription linking FoxM1 transcriptional activity to the Ras/Raf/MEK/ERK1/2 pathway. FoxM1 may also be connected to the PI3K/AKT pathway, as overexpression of activated AKT can increase FoxM1 expression (128).

FoxM1 may be suitable as a predictive marker in ENDO-R. Overexpression of FoxM1 in ERα-positive breast cancer was found to correlate with worse prognosis of TAM-treated patients (94, 126). Interestingly, a gene signature linked to the protein 14-3-3ζ, a FoxM1 regulator, is also associated with unfavorable prognosis of TAM-treated patients (129) suggesting that a 14-3-3ζ-FoxM1 axis can drive ENDO-R.

FoxM1 might be targeted through 14-3-3ζ, whose activity can be inhibited by small molecules, such as FOBISIN101, or by the peptide inhibitor R18 (130). R18 was found to strongly support the apoptotic effect of TAM on MCF-7 cells (131).

### Hypoxia-Inducible Factor 1/2α

Hypoxia stabilizes HIF-1α and HIF-2α proteins allowing them to initiate transcription of numerous genes engaged to ensure survival under hypoxic conditions (132). In cancer, also non-physiological activation of these transcription factors occur (133). HIF-1α and HIF-2α are involved in tumor progression (134). Among others, they promote metastasis and cancer stem cell activity (*Cancer Stem Cells*).

Overexpression of HIF-1α or HIF-2α was found to desensitize MCF-7 cells to FULV (135, 136). Likewise, exposure to hypoxia reduced ERα expression and FULV sensitivity of various ERα-positive breast cancer cell lines (136, 137). Also, FULV-R MCF-7 cells showed higher expression of HIF-2α, but not HIF-1α, and could be sensitized to FULV by inhibition of HIF activity.

One target of HIF-2α is EGFR, which has been linked to anti-estrogen resistance. EGFR can also feedback on HIF-2α (136). Furthermore, HIF-2α expression is driven by FoxA1 (107) linking HIF-2α and EGFR to FoxA1.

Interestingly, HIF and ERα share many genes that they can transcriptionally activate (138). Of these, sodium-dependent neutral amino acid transporter 2 (SNAT2) has been linked to FULV resistance. SNAT2 is a transmembrane transporter for short chain neutral amino acids, such as glutamine, and an amino acid sensor (139, 140). When overexpressed in MCF-7 cells, SNAT2 induces FULV resistance *in vitro* and *in vivo* (138). FULV-R or TAM-R MCF-7 cells can use glutamine instead of glucose for maintaining metabolism (141), which may play a role in SNAT’s ability to induce FULV resistance. SNAT2 overexpression was associated with worse outcome in luminal B-type, but not in luminal A-type cancers (138).

The activation of HIF also leads to a disconnect between glycolysis and the tricarboxylic acid cycle, whose maintenance becomes then dependent on glutamate (142). Hence, when HIF is activated, glutamine metabolismus is gaining importance in cancer’s energy generation. Therefore, endocrine resistant breast cancer with high HIF activity may be responsive to drugs interfering with glutamine metabolism. A promising druggable target is glutaminase (GLS) which converts glutamine to glutamate (143). The GLS-inhibiting drug CB-839 is now tested in clinical trials (142). In one study, it is combined with paclitaxel to treat TNBCs.

### Nuclear Factor κB

The NFкB pathway has been linked to oncogenesis (144) and to ENDO-R (145). The NFкB family of transcription factors include NF-κB1 (p50), NF-κB2 (p52), RelA (p65), RelB and c-Rel, which homo- or heterodimerize to interact with specific DNA binding sites. Upon phosphorylation of the NFкB regulator IκB (inhibitor of NFкB) by IKK (IκB kinase) the NFкB protein is released from the inhibitory complex and translocates to the nucleus to regulate transcription (146).

Being a strong activator of cyclin D1 synthesis (22), NFкB may replace ERα in stimulating proliferation when ERα activity is impaired. In a number of FULV-R MCF-7 sublines, increased NFкB (p65, RelB) activity has been noted, whose inhibition resulted in growth-suppressive effects (147–150). NFкB has also been found to prevent apoptosis of FULV-R MCF-7 cells (150).

In FULV resistance induced by mesenchymal stem/stromal cells (MSCs) or carcinoma-associated fibroblasts (CAFs) the atypical IκB protein B-cell lymphoma-3 (Bcl-3) plays a role, whose expression is associated with poorer survival of TAM-treated patients (90). Bcl-3 can activate NFкB-dependent transcription by binding to transcriptionally repressive p50/p50 and p52/p52 homodimers and “convert” them to activators (151). Bcl-3 is a growth-stimulatory factor in cancer cells (152, 153). It may partially act as such by upregulating the expression of c-Myc (154, 155), a proliferation-inducing protein which may contribute to ENDO-R (141, 156) and by stimulating the expression of sex determining region Y-box 2 (Sox2) (157), a stem cell protein involved in drug resistance (158). In addition, Bcl-3 downregulates the expression of selenoprotein P, plasma 1 (SEPP1) and kelch-like 4 (KLHL4) (90), two genes whose mRNA levels inversely correlate with relapse-free survival of TAM-treated patients. Interestingly, KLHL4 has recently been reported to bind p53 to increase the expression of the cell cycle inhibitor p21 (159). Hence, part of Bcl-3’s growth-stimulatory activity may be based on its suppressive effect on KLHL4 expression.

A number of drugs interfering with the NFкB pathway have been developed (160). some of which are used in clinical trials (161). For instance, the anti-alcoholismus drug disulfiram, which also inhibits NFк;B activity, is tested in a phase II trial of patients with a Her2-negative BC.

### X-Box Binding Protein-1

Unfolded protein response (UPR) is activated in the event of endoplasmic reticulum stress (162). UPR is important for ENDO-R, as it is able to act as prosurvival mechanism by eliminating endoplasmic reticulum stress and by re-installing metabolic homeostasis (163). XBP1 is a key transcription factor involved in regulating UPR and is activated by UPR. Upon UPR initiation, XBP1 is activated by unconventional cytoplasmic splicing resulting in the conversion of XBP1 mRNA coding for the unspliced XBP1(U) form to the mRNA encoding spliced XBP1(S) form. In contrast to the XBP1(U) protein, the longer XBP1(S) protein harbors a transactivation domain allowing XBP1(S) to activate transcription through CREB responsive elements. One important target gene of XBP1(S) is *esr1*, the gene coding for ERα, another the gene encoding the NFкB transcription factor p65/RelA (164). Overexpression of XBP1 renders MCF-7 cells more resistant to FULV (165), while its depletion reduces cell growth of FULV-R MCF-7 cells by inducing apoptosis (164), likely caused by reduced expression of XBP1-regulated anti-apoptotic protein Bcl-2 (165). Higher ratio of XBP1(S)- to XBP1(U)-mRNA correlates with worse prognosis of patients with ERα-positive BC, while XBP1(U) expression alone predicts better survival (100).

The importance of UPR for drug resistance has planted the idea of inducing an overload of ER stress (166). This could be achieved by certain nanoparticles or by the proteasome inhibitor bortezomib, the latter being already used to treat certain haematopoietic cancers.

### Y-Box Binding Protein 1

The transcription factor YB-1, a so-called cold-shock protein, is involved in cellular stress responses (167). By binding to the ERα protein and interfering with its activity (168, 169) and by upregulating the expression of EGFR and Her2 (168, 170, 171), YB-1 induces a shift from ERα- towards EGFR/Her2-driven gene expression. In line with this, in primary BCs, YB1 expression correlates with the expression of EGFR and Her2 and inversely with that of ERα and PR (170, 172, 173). Higher YB-1 expression is associated with poorer prognosis in BC (174–178) and predicts a worse outcome of TAM-treated patients (101).

Its role in ENDO-R is further supported by the finding that ectopically expressed YB-1 desensitizes MCF-7 and T47D cells to FULV and TAM (179, 180). Lapatinib counteracts the FULV-de-sensitizing effect of YB-1 confirming the involvement of EGFR and Her2. Silencing of YB-1 reverses the switch from ERα to Her2 expression and re-sensitizes cells to anti-estrogens. Interestingly, in FULV-R cells, YB-1 expression is not upregulated, but its phosphorylation at Ser102 is increased (180). P-Ser102 modified YB-1 has been shown to foster anchorage-independent growth and radiation resistance of BC cells (181, 182). Ser102 can be phosphorylated by AKT, p70S6K, and ribosomal S6 kinase (p90RSK) (180). As shown with MCF-7 and ZR75-1 cells, FGFR2-dependent signaling increases the interaction between YB-1 and ERα (169), suggesting also a link between YB-1 and FGFR2.

Interference with YB-1 activity is possible by the novel multikinase inhibitor TAS0612, which targets AKT, p70S6K, and p90RSK and thereby prevents YB-1 phosphorylation at Ser102 and its subsequent transport into the nucleus (180). TAS0612 was shown to efficiently suppress growth of Fulv-R BC cells *in vitro* and *in vivo*.

## Chromatin Accessibility

Chromatin accessibility is defined by the ability of DNA-binding factors to access chromatin DNA, which is highly compacted by its interactions with histones and other chromatin-binding factors (183). Chromatin accessibility is vital to active transcription. Only 2-3% of the chromation contains accessible DNA to which 90% of the TFs bind. Besides pioneer TFs, chromatin remodeling complexes, such as switch mating type/sucrose non-fermenting (SWI/SNF), histone modifiers, histone readers and mediators play an important role in opening up chromatin (75, 76). Post-transcriptional modifications (PTMs) of histones play a key role in regulating chromatin accessibility (184). PTMs are regulated by “writing” enzymes that add a modification and “erasing” enzymes that remove a modification (185). For instance, histone acetyl transferases (HATs), such as CBP, acetylate histones, thereby promoting transcription, while histone deacetylases (HDACs) deacetylate histones, thereby repressing transcription. PTMs can be recognized by histone readers. Bromodomain histone readers, such as bromodomain-containing protein 4 (BRD4), recognize acetylated histones (186). The bromodomain and extraterminal (BET) family of bromodomain histone readers has recently gained attention as a potential target in cancer therapy.

Long-term repression of transcription can be achieved by DNA methylation, leaving an epigenetic mark that can be transmitted to daughter cells. Abnormal *de novo* DNA methylation in tumorigenesis prevent the activation of key genes involved in terminal differentiation and thereby in inhibition of proliferation (187).

Resistance to FULV, TAM or AI is accompanied by changes in histone PTM and DNA methylation patterns indicating that resistance to these drugs are accompanied by epigenetic reprogramming (188–190).

### AT-Rich Interaction Domain 1A

Among the genes required for the anti-proliferative effects of FULV and TAM is ARID1A, a factor of the SWI/SNF complex BAF (28). It is recruited by FoxA1 to FoxA1/ERα-regulated genes and in turn attracts HDAC1, thereby blocking ERα-depending transcription (**Figure 2**). Loss of ARID1A leads to increased histone 4 acetylation and recruitment of BRD4 to these genes. This allows that these genes can be transcribed even though anti-estrogens are present, which eventually results in FULV and TAM resistance (28).

Higher expression of ARID1A correlates with good prognosis in BC (89). However, mutations in ARID1A gene, found in 5% of primary and 12% of metastatic BCs, are associated with unfavorable prognosis (28). BET-inhibitors, available for therapy of cancer patients (191), may be useful tools to counteract ENDO-R caused by ARID1A dysfunction.

### HDACs

Based on their homology to yeast deacetylases, four classes of human HDACs are distinguished: class I, IIa, IIb. III and class IV (192). Originally identified as enzymes that deacetylase histones, HDACs were later found also to modify non-histone proteins. Class I HDACs (HDAC1, -2, -3, and -8) are primarily located in the nucleus and engaged in histone deacetylation. By removing acetyl group from lysines, histones become more positively charged, which strengthens the interaction with the negatively charged DNA. This leads to higher compaction of the chromatin, which is then less available for transcription (185). HDACs are typically recruited by transcriptional repressors.

In ERα-negative breast cancer cells, HDAC1 contributes to the inactivation of the *esr1* promoter (193). In addition, independent of its histone-regulating function, HDAC1 binds directly to the ERα protein, thereby further suppressing ERα activity (194). Subsequently, suppression of HDAC activity by an HDAC inhibitor (HDACi) results in re-occurrence of the ERα protein in ERα-negative cells (195, 196). Furthermore, inhibition of HDAC3 was shown to reduce the formation of FULV-R MCF-7 colonies (197). Also, knock-down of HDAC2 was found to strongly increase the sensitivity to TAM (198). Treatment of TAM-R MCF-7 cells with HDACi was reported to induce apoptosis as well as autophagy and to reduce cellular growth *in vitro* and *in vivo* (199–201). It has been speculated that alterations in the expression of ERα co-repressors, such as nuclear co-repressor (NCoR) 1 and 2, may play a role in the cytotoxic effect of HDACi on TAM-R BC cells, as these co-repressors recruit HDACs (202). Loss of such co-repressors may lead to an epigenetic imbalance of ERα-driven gene activity. Importantly, NcoR1 is lost in more than half of all ERα-positive BCs.

In a phase III trial, patients who relapsed on endocrine therapy show a survival benefit when treated with the HDACi tucidinostat in addition to the AI exemestane (96). Hence, there is evidence that HDACs are involved in ENDO-R.

### Non-Canonical Histone Variant H2A.Z

Histone variants replace canonical histones at certain places of the chromatin, particularly in transcriptionally active regions of the genome, and thereby locally influence epigenetics (203). Histone variants may allow higher rates of nucleosome turnover and may improve chromatin remodeling at active promoters and enhancers.

There is growing evidence that cancer cells misuse histone variants to foster their proliferative activity. In breast cancer, the mRNA expression of the histone variant H2A.Z correlates with the mRNA levels of cell cycle proteins, including cyclins (204). H2A.Z may be of particular importance for ERα-driven breast cancer (203). Of the two H2A.Z proteins, H2A.Z.1 and H2A.Z.2, H2A.Z.1 is regulated by ERα through an ERE site in its gene *h2afz*. Furthermore, H2A.Z is recruited to hypomethylated DNA at ERα-active enhancers (205) and is important for estrogen-dependent ERα activity at FoxA1/ERα binding sites (206). Interestingly, ectoptic expression of H2A.Z was shown to increase MCF-7 cell proliferation in the absence of estrogen or in the presence of TAM suggesting a potential role of this protein in ENDO-R (204). Overexpression of H2A.Z is associated with poor outcome in BC (95).

### DNA Methylation

Compared to the MCF-7 parental cell line the DNA methylation pattern is different in FULV-R MCF-7 sublines (33, 189, 207, 208). Both altered hyper- and hypomethylation of promoters and enhancers were found to coincide with FULV resistance. Hypermethylation of promoters in the anti-estrogen-resistant sublines was linked to either a higher expression of DNA methyltransferase (DNMT) 3B or DNMT1 (207, 209). In FULV-R MCF-7 cells, promoter A of the *esr1* gene is one of the hypermethylated promoters giving rise to strongly reduced ERα expression (208). In contrast, in FULV-R T47D cells, loss in promoter A activity did not coincide with hypermethylation. PTEN is another gene whose promoter can be highly methylated in anti-estrogen resistant MCF-7 cells (209).

In TAM-R MCF-7 cells, hypermethylation was predominantly found in enhancers (189). Of these enhancers ~20% were ERα-responsive, of which approximately half contained FoxA1 binding sites. Importantly, methylation in the ERα-responsive enhancers significantly reduced ERα binding and the expression of the enhancer-driven genes. A higher methylation status in these enhancers was found to be linked to a higher risk of relapse on TAM treatment. Methylation of the ERα-responsive enhancers seems also to play a role in regulating ERα transcriptional activity in the different BC subtypes. The highest median methylation of ERα-responsive enhancers was found in the ERα-negative subtype, whereas it was lowest in luminal A tumors (189).

## Cancer Stem Cells (CSC)

There is a great body of evidence that a minor population of cells with stem-like activities, CSCs, are responsible for BC growth initiation and progression (210, 211). To identify CSCs in BC, several markers have been established, among them CD44, CD24, CD133 and aldehyde dehydrogenase 1 (ALDH1) (212, 213). By being multidrug resistant (214) and by showing low expression of ERα (98, 215) CSCs are highly likely to escape endocrine treatment. If so, anti-estrogens, by eradicating non-CSCs while leaving CSCs alive, would increase the proportion of the CSC population. Indeed, treatment with FULV or TAM has been found to enrich the CSC fraction in the MCF-7 cell line (98, 216). Also, high expression of ALDH1 is associated with failure of ERα-positive BCs to respond to TAM (98). As shown with MCF-7 cells in mouse xenografts, one subpopulation of CSCs (CD133^hi^/CD44^low^) may be of particular importance in FULV-R (217). In BC, higher expression of CD133 correlates with lower response rates to chemotherapy (218) suggesting a general role of CD133 in drug response.

CSC activity is maintained by a number of transcription-regulating factors, such as cleaved fragments of the Notch pathway, β-catenin activated by the Wnt pathway and epithelial-to-mesenchymal transition (EMT)-inducing-TFs (211).

### The Notch Pathway

The Notch signaling pathway, important for the maintenance of CSC activity in BC (211), has been linked to ENDO-R (219, 220). To stimulate signaling through the Notch pathway, a Notch receptor interacts with a ligand of the Delta-Serrate-Lag2 (DSL) family, such as JAG1, presented by a neighboring cell. This leads to a γ-secretase-dependent cleavage of the Notch protein resulting in the Notch fragment Notch intracellular domain (NICD) (221, 222). Imported into the nucleus, NICD induces transcription of genes, such as hairy and E(spl (Hes), engaged in regulation of cell fate decisions.

In MCF-7 and T47D cells, Notch1 and Notch4 activities are negatively regulated by estrogen-activated ERα (91, 223). The ERα-dependent repression of the *notch4* gene involves the transcriptional repressor death domain associated factor 6 (DAXX), a protein stabilized by ERα. In turn, DAXX recruits DNMT1 to the *notch4* promoter leading to DNA methylation. Besides Notch4, DAXX also down-regulates other stemness-relevant genes, including ALDH1A1, thereby causing the tumor-initiating capacity of BC cells to decline. Importantly, higher expression of DAXX correlates with more favorable outcome of patients who received endocrine treatment (91).

Consistent with the repressive effect of ERα on Notch activity, inhibition of ERα in MCF-7 and T47D cells by FULV or TAM increases Notch pathway activity, particularly the activities of Notch 3, 4 and JAG1 (91, 97, 98, 224–226). Furthermore, activation of the Notch pathway renders MCF-7 cells resistant to TAM, which coincides with higher NICD levels of Notch1, 3 and 4 (224). Moreover, higher Notch activity predicts worse outcome in ERα-positive BCs (97, 98).

The link between the ERα and Notch pathways may be more complex. One study shows that Notch1 and JAG1 are involved in ERα expression (227) and that silencing of either protein resulted in a loss of luminal marker genes and a gain in basal-like marker genes. Another study suggested an ERα-driven cross-talk between non-CSCs and CD44^+^/Epcam^+^/CD24^-^ -CSCs, by which the Notch pathway is activated to increase the CSC population (228).

Inhibitors of the Notch pathways, such as γ-secretase inhibitors, are tested in breast cancer trials (220) and may be suitable tools to treat Notch-dependent ENDO-R.

### The Wnt Pathway

The Wnt pathway is an important pathway in mammopoiesis, involved in mammary stem cell regulation and cell fate decisions (229). Its deregulation can lead to BC. In the canonical Wnt pathway, a Wnt ligand interacts with the Wnt receptor Frizzled, which in concert with its co-receptor low-density lipoprotein receptor-related (LRP) leads to stabilization of the protein β-catenin in the cytoplasm (230). Translocated to the nucleus, this key effector of the Wnt pathway drives transcription by interacting with the transcription factor T cell factor/lymphoid enhancer-binding factor. Among the target genes are the EMT-TFs Twist and Slug. Through a different pathway Wnt/Frizzled interaction leads to increased ATF-2/c-Jun activity (230), two factors of the AP-1 family discussed to be involved in ENDO-R (*AP-1*).

In FULV-R and TAM-R MCF-7 sublines, expression of Wnt pathway components, including β-catenin, are increased (33). Also, overexpression of β-catenin in MCF-7 cells decreased their sensitivity to FULV (231). Higher β-catenin cytosolic and/or nuclear abundance have been linked to poor survival in BC (232). Since this was found for all BC subtypes, it may simply reflect a higher degree of CSC activity.

### EMT-TFs

EMT is an essential process in embryonal development and wound healing allowing stationary cells to switch to a migrating phenotype (233). This is caused by a set of EMT-TFs, such as Twist, Slug and zinc-finger E-box binding homeobox 1 (ZEB1). EMT can bestow cancer cells stem cell features and convert them to CD44^+^/CD24^-^ CSCs (234). EMT may not lead to a fully formed mesenchymal phenotype, but may give rise to intermediate states, now called quasi-mesenchymal phenotypes (235). Quasi-mesenchymal CSCs may be of particular importance for cancer progression.

Overexpressed in MCF-7 and T47D cells, Twist was shown to bind to the *esr1* promoter and to inhibit ERα expression, leading to estrogen-independent proliferation and FULV and TAM resistance (236). Twist-induced suppression of *esr1* transcription coincided with DNA methylation, caused by the Twist-recruited DNMT3B. Like Twist, ZEB1 induces *esr1* promoter hypermethylation and ENDO-R (237), while ZEB1 downregulation increases FULV sensitivity (238). In FULV-R and TAM-R MCF-7 sublines, primarily Slug was found to be overexpressed (239).

Clinically, higher expression of Twist, Snail or Slug was found to be associated with a higher probability to relapse in ERα-positive BCs (99). Twist and Snail combined were even more powerful in predicting the risk of relapse than each protein alone. However, it remains unclear whether the link between EMT-TF expression and poor clinical outcome is based on the down-modulatory effects of EMT-TFs on ERα signaling or on their ability to promote cellular migration.

## Conclusions and Future Perspectives

Currently, the PI3K/AKT/mTOR pathway and CDK4/6 are the prime targets to manage endocrine resistance. However, inhibitors against these targets may fail. For instance, ENDO-R resulting from Her2 mutations or FGFR1 amplification are also resistant to the CDK4/6 inhibitor palbociclib (60, 240). Hence, there is a need for a list of biomarkers predicting the responses to the currently used inhibitors. Unraveling the nuclear mechanisms involved in ENDO-R may lead to the discovery of additional biomarkers which may help to optimize treatment of endocrine resistant BCs.

Furthermore, by exploring the nuclear mechanisms that allow escape from endocrine treatment new druggable targets may come to light. However, the diversity of nuclear mechanisms leading to ENDO-R requires additional diagnostics to clarify which nuclear changes are responsible for the observed resistance. One such recently identified promising target is the histone PTM reader BRD4 (28), whose activity can be blocked by BET-inhibitors already available for therapy of cancer patients (191). HDACi, also available for treatment of cancers (192), may be an option to overcome HDAC-dependent suppression of estrogen-driven transcription (96).

Transcription factors are more difficult to target, as they usually lack enzymatic activity. However, drugs can interfere with these factors indirectly, for instance, by blocking enzymes responsible for their activation or by inhibiting their interactions with essential co-factors. Transcription factors that are druggable through such an approach include YB-1, NFкB, Notch and ERRα. YB-1 activity can be suppressed by blocking the kinases that catalyze an essential activating phosphorylation event (180). NFкB can be kept in an inactivated state by IKK inhibitors or by the anti-alcoholismus and anti-cancer drug disulfiram, which seems to interfere with an essential proteolytic step in the NFкB pathway (161). Notch activity can be blocked by γ-secretase inhibitors, which are already used in the clinic to treat cancers (241). As the Notch pathway is also important for maintaining the CSC population in BC, γ-secretase inhibitors may also counteract the rise of the CSC population during endocrine treatment. ERRα inhibitors, developed to treat diabetes (102), interfere with the interaction of ERRα with its co-factor SRC.

Transcriptional activities could also be controlled by interfering with certain miRNAs. For instance, by blocking miR221/222 β-catenin-dependent transcription FULV resistance can be suppressed (231).

Thus, understanding the nuclear mechanisms involved in ENDO-R may help to dissect those patients who benefit most from treatment with PI3K/AKT/mTOR pathway and CDK4/6 inhibitors and, additionally, may allow the identification of new druggable targets.

## Author Contributions

The author confirms being the sole contributor of this work and has approved it for publication.

## Conflict of Interest

The author declares that the research was conducted in the absence of any commercial or financial relationships that could be construed as a potential conflict of interest.

## Publisher’s Note

All claims expressed in this article are solely those of the authors and do not necessarily represent those of their affiliated organizations, or those of the publisher, the editors and the reviewers. Any product that may be evaluated in this article, or claim that may be made by its manufacturer, is not guaranteed or endorsed by the publisher.
